# Sub-8-minute cardiac four dimensional flow MRI using kat ARC and variable density signal averaging

**DOI:** 10.1186/1532-429X-17-S1-Q36

**Published:** 2015-02-03

**Authors:** Peng Lai, Ann Shimakawa, Joseph Y Cheng, Marcus T Alley, Shreyas Vasanawala, Anja C Brau

**Affiliations:** 1 Global MR Applications and Workflow, GE Healthcare, Menlo Park, CA, USA; 2 Radiology, Stanford University, Palo Alto, CA, USA

## Background

Recently, 3D CINE phase-contrast MRI (4D Flow) has demonstrated potential for quantitative measurement of blood flow of the cardiovascular systems in the entire thoracic trunk [1]. However, the needs of 4D flow for high resolution, large volume coverage & respiratory gating render very long scan time. This work intended to improve the data acquisition efficiency of free-breathing cardiac 4D flow.

## Methods

**Figure 1:**
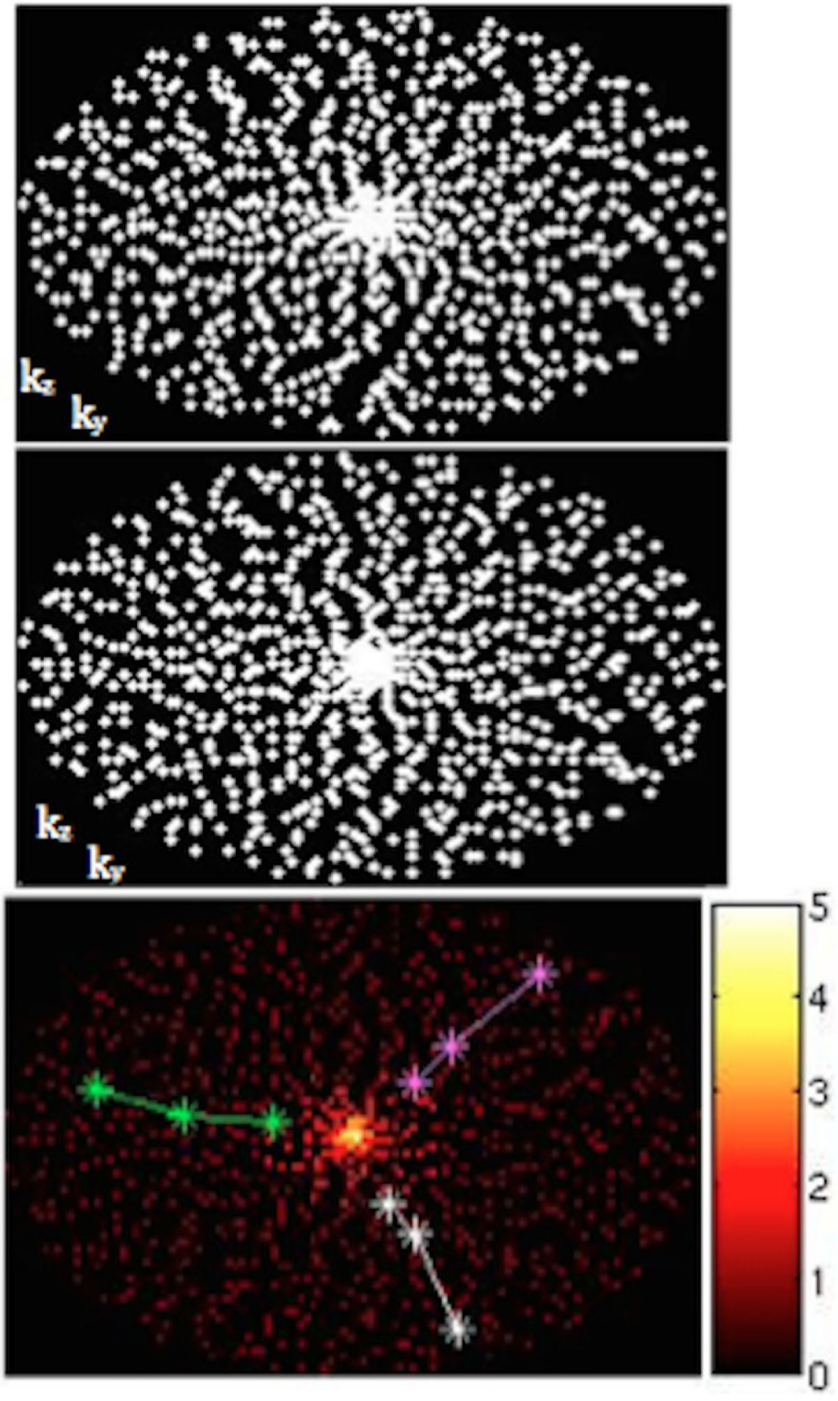
VDR kt sampling at t=8 (upper) & 9 (middle). Bottom: variable density NEX factor. 3 colored lines indicate 3 successive trajectories.

k-t Acceleration: kat ARC [2], a spatiotemporal-correlation-based autocalibrating parallel imaging method, was used for accelerating 4D flow. The acquisition & reconstruction were optimized for high acceleration. As shown in Figure 1, data was collected with a variable density random (VDR) k-t sampling scheme [3] to improve overall reconstruction accuracy and reduce coherent artifacts.

In reconstruction, a static tissue removal scheme [4] was adapted for 4D flow to reduce residual aliasing artifacts. Specifically, from initial view-sharing reconstruction, static tissues (e.g. chest wall, spine, etc) with no flow or motion were identified. Next, static tissue signal was removed from the original data to reduce aliasing in undersampled data. After kat ARC reconstruction of dynamic signal, the static tissue signal was added back to generate the final image.

Variable density signal averaging: signal averaging of multiple excitations is a commonly used strategy for free-breathing MRI, but it requires multiple-fold increase in scan time. This work used a variable density number of excitations (NEX) scheme for improving scan efficiency. Figure 1 demonstrates the acquisition scheme. The NEX factor is the highest at center k-space and decreases toward outer k-space for an optimal compromise between motion artifacts and scan time. Furthermore, a radial golden angle vieworder was used to minimize the adverse effects of residual motion artifacts [5].

To evaluated the proposed method, 5 healthy adult volunteers were scanned on GE 3T (MR750) using 32-channel cardiac coil without contrast. 4D flow MRI was performed covering the entire chest. Imaging parameters were: 380x250 mm2 FOV, 2x2mm2 resolution, 72 slices, 2.5mm thickness, 60ms temporal resolution, 8× acceleration.

## Results

**Figure 2:**
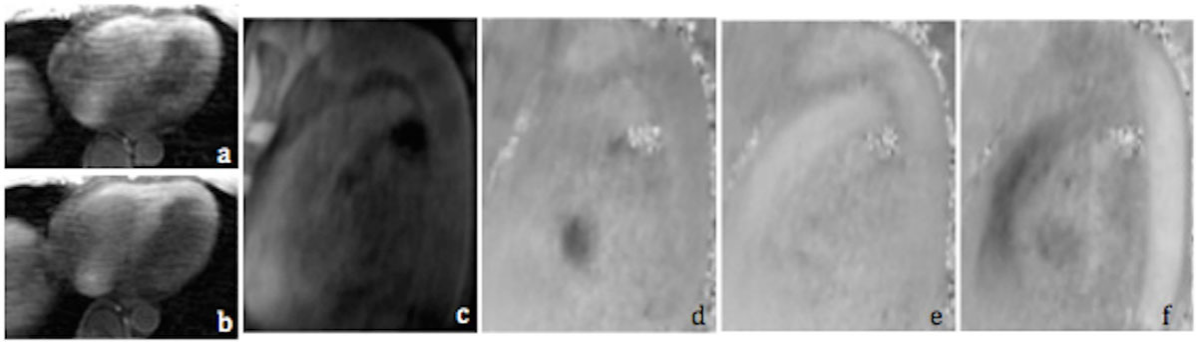
Comparison of acquisition without (a) and with (b) variable density NEX. Magnitude (c), LR (d), AP (ef) and SI (f) flow of descending aorta at a reformatted plane.

As shown in Fig. 2, compared to acquisition without variable density NEX (b), the proposed acquisition scheme (a) effectively reduced respiratory motion artifacts. On all subjects, we were able to obtain 4D flow images with only minor residual motion artifacts and perform offline visualization and measurement of blood flow in an arbitrary reformatting. The average scan time was ~6.5 & ~5.5 min for scans with & without variable density NEX, respectively.

## Conclusions

This work developed a variable density NEX scheme for cardiac 4D flow MRI with high scan efficiency. Combined with kat ARC, we were able to perform whole-chest 4D flow under 8min. Further clinical evaluation will be performed to evaluate the proposed 4D flow method.

## Funding

N/A.
